# Barriers, motivations, and understanding: A qualitative study on the acceptability of an academic weight management program

**DOI:** 10.1371/journal.pone.0351217

**Published:** 2026-06-10

**Authors:** Yongjia Deng, Kristen Moore, Patricia Galanti, Laura Davisson, Nasser Alrayyes, Cristhian Perez, Patricia Dekeseredy, Treah Haggerty

**Affiliations:** 1 West Virginia University School of Medicine, Morgantown, West Virginia, United States of America; 2 West Virginia University School of Medicine, Department of Medicine, Morgantown, West Virginia, United States of America; 3 WVU Medicine, Medical Weight Management, Morgantown, West Virginia, United States of America; 4 West Virginia University School of Medicine, Department of Neurosurgery, Morgantown, West Virginia, United States of America; 5 West Virginia University School of Medicine, Department of Family and Community Medicine, Morgantown, West Virginia, United States of America; McGill University Faculty of Arts, CANADA

## Abstract

**Introduction:**

Obesity is a critical health issue, and West Virginia is a state with an exceptionally high obesity rate. There are evidence-based medical guidelines for treating obesity utilized by medical weight management programs. The primary purpose of this study is to gain a comprehensive understanding of the acceptability of an academic medical weight management program that utilizes guidelines-based treatment for obesity.

**Methods:**

This study employs a qualitative descriptive approach. Semi-structured interviews were completed with 20 participants enrolled in an academic medical weight management program. Interviews were completed and transcribed. Themes were identified through qualitative content analysis.

**Results:**

Twenty participants enrolled in a medical weight management clinic participated. Five main themes emerged during analysis of the interview content: 1) satisfaction with clinical and support staff, 2) desire for additional touchpoints with clinical staff, 3) perception of non-judgmental support, 4) perception of support by the clinic following policy changes affecting treatment coverage, and 5) participation due to intrinsic motivation.

**Conclusion:**

Patients enrolled in medical weight management clinics exhibit a wide diversity of motivations for their desired weight loss, but commonly face a variety of challenges to their care, including unreliable insurance coverage and social stigma related to obesity. However, the resulting themes identify acceptable approaches for medical weight management clinics such as increase touch points, supportive clinical staff who provide non-judgmental support, and an understanding of participants intrinsic motivations.

## Introduction

The increasing prevalence of obesity in the United States is a major health crisis and may contribute to preventing gains in national life expectancy [1]. Obesity has also led to related healthcare expenditures in the hundreds of billions of dollars annually [1]. This is particularly important in West Virginia, as the state leads the nation in obesity with a rate of 41.2 percent with adult women having a rate of 42.2 percent and men having a rate of 40.1 percent [2]. Many factors come together to produce West Virginia’s high obesity rate, including the rural nature of the state and its relative poverty, both of which have been linked with obesity [3,4]. As of 2023, West Virginia has a poverty rate of 16.7 percent, which is the fourth highest in the country [5] and the median household income is the second lowest in the country [6]. Additionally, 64 percent of its population resides in rural areas.

In response to this crisis, West Virginia University Medicine (WVU Medicine) set up several specialized medical services including a bariatric surgery program. Subsequently, WVU Medicine started an medical weight management program in 2019 [7]. The WVU Medicine Medical Weight Management (MWM) clinic is a comprehensive medical service aimed at treating obesity using nonsurgical methods and includes access to a multidisciplinary team consisting of healthcare practitioners, nursing staff, dietitians, behavioral medicine, and exercise specialists [7,8]. Demand for the clinic’s services has been exceptionally high, with patients often spending a year on the waitlist before their first appointment.

However, not much is known about patient perceptions of acceptability in taking part in an academic medical weight management clinic. Patients at the clinic typically receive a generic feedback survey after each visit. However, those surveys are not tailored to the unique aspects of MWM and are generally not completed by the majority of patients. To better serve its patient population, we conducted an acceptability study to identify the facilitators and barriers of the MWM clinic.

Acceptability studies are a type of study designed to determine how a program or intervention meets the needs of its target population. Similar studies on weight management programs have been performed in the past. Those studies generally examined questions such as the motivations behind joining a program, the perceived advantages and disadvantages of certain programs, acceptable and unacceptable factors, and perceived barriers to achieving weight management results [9]. However, many of these studies were on non-medical weight loss programs. Additionally, very few of those studies broke down participant quotes by demographic factors such as gender or socioeconomic status [9]. The primary purpose of this study is to gain a comprehensive understanding of the acceptability of an academic medical weight management program with a predominantly rural patient population within the United States.

## Methods

Participants: This qualitative research study was completed with participants from an academic medical weight management clinic. Inclusion criteria included that participants were 18 years old or older and had been seen in the clinic from January 1, 2023, to December 31, 2023. Participants were required to converse easily in English and have the ability to complete interviews through a virtual platform. Patients were not eligible for participation in the study if they were below the age of 18 or if they had any cognitive impairments that would prohibit them from consenting to participate in the study.

### Procedures

Participants seen in the clinic on the identified dates were delivered an electronic medical record portal message inviting them to participate in the study. The portal message included information about the study and a link for virtual consent. This portal message was sent out to 8,395 participants; 292 of these participants responded that they were interested in interviewing. Participants were scheduled for an interview after consent procedures were complete. One portal message was sent in September 2024 and participants were enrolled from September 2024 till November 2024. Participant recruitment for the study took place from 9/1/2024 until 11/30/2024. Participants were contacted, reviewed for inclusion and exclusion criteria, and consented for inclusion in the study in order as they responded to the portal message desiring to take part in the study. Interviews were conducted in a private location via virtual conferencing through Zoom, with transcription provided at the time of the interview automatically by the platform. Participants were generally selected on a first-come, first-serve basis. When possible, efforts were made to select a diverse set of participants that represented the local population; additional efforts were made to select for male participants after it was noticed that participants were disproportionately female initially. The West Virginia University Institutional Review Board approved the protocol for this study (#2309858370).

### Data collection

Semi-structured interviews were completed with 20 participants. Interviews were completed by two male co-investigators (YD and NA). An interview guide was initially created by multiple members of the study team (TH, YD, NA, and CP). The interview guide was informed by the Consolidated Framework for Implementation Research [10,11] (CFIR) and included constructs from four of the five CFIR domains including intervention characteristics, characteristics of the individual, integration and sustainability, inner setting, and outer setting. Interview times varied from participant to participant with the average interview time being 24 minutes and ranging from 13 to 32 minutes. Immediately after the interview the transcripts were reviewed for accuracy of the data, and identifiers were removed.

### Data analysis

Content analysis, as described by Hsieh and Shannon [12], was used to systematically identify patterns and themes within the interview transcripts. Three co-investigators (TH, YD, and NA) independently reviewed all de-identified transcripts to achieve immersion in the data and to develop an initial understanding of participant perspectives. During this initial review, investigators generated preliminary codes derived inductively from the data, which were compiled into an initial codebook. Transcripts were then re-reviewed and coded using this codebook. Through an iterative process of discussion, reflection, and constant comparison, codes were refined, merged, or expanded to improve clarity and consistency. Discrepancies in coding were resolved through consensus among the investigators. Following coding, related codes were grouped into higher-order categories, and emergent themes were identified. These themes were further refined through group discussion to ensure they accurately represented the data and captured shared patterns across participants.

## Results

**Fig 1:**
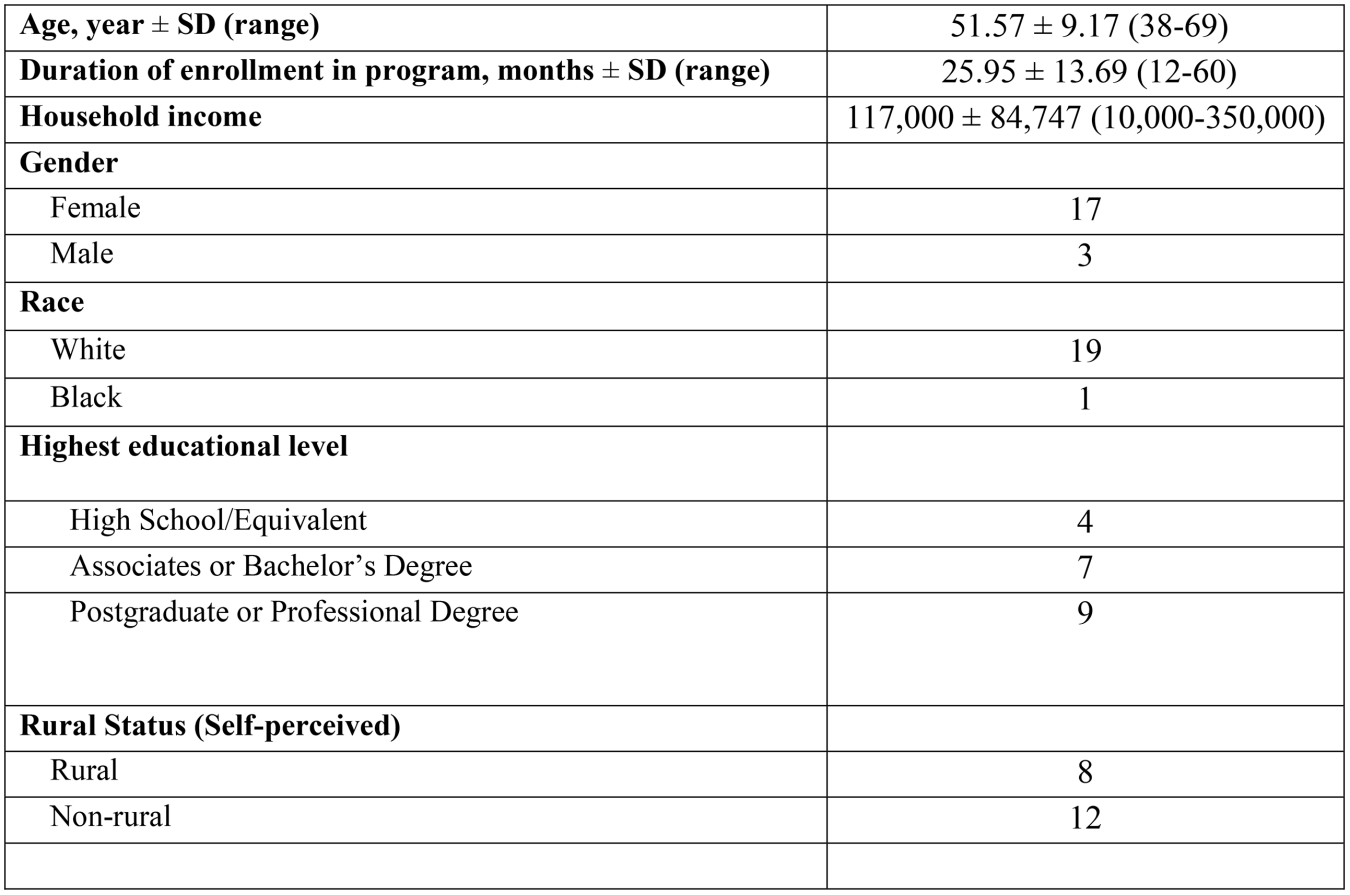
Participant demographics.

All participants attend a single multidisciplinary academic obesity medicine clinic at West Virginia University Medicine [7,8]. Findings from this analysis resulted in five themes Fig 1.

### Satisfaction with clinical and support staff

Participants reported satisfaction with taking part in the Medical Weight Management clinic. With obesity being a chronic and relapsing medical condition, many participants reported trying other methods of weight management in the past. Participants felt dissatisfied due to weight regain however, their time in this medical program satisfied their need for appropriate obesity care.

“No, it was the staff. The staff from the physician to the midlevel providers (sic) were very welcoming and made sure that if I didn’t feel as though my needs were being met through telemedicine, what other ways could we try to manage like meeting in person, if that was possible, or meeting more frequently.” -Participant 1 (38 Year Old White Female; Non-Rural)

Participants were overall satisfied with the care delivery methods in the clinic. Participants noted that they were able to use telemedicine when desired. However, some participants were doing telemedicine and felt that they would be better served with in-person visits.

“I was able to do video visits, so it was very convenient for me… The follow-up visits are very convenient and effective, so.” -Participant 4 (44 year old white female; non-rural)

### Participants desire more touchpoints with the clinic

Many participants desired more frequent interactions with health practitioners at the clinic. The primary reason stated for wanting additional touchpoints was for an additional sense of accountability. Many of the participants felt that more frequent visits would provide this accountability and potentially improve their weight loss progress.

“Part of the program that I don’t find helpful is that it’s so long between appointments. It took almost a year to get in! It was that far backed up as far as getting into the program, and then I don’t know what my expectations were in the beginning, but I think I expected it to be more than once every couple of months, or every 3 months, that you actually get into the office to see someone.” “More opportunities to interact … (would help with) keeping you right, keeping you working all the time.” – Participant 3 (68 year old white male; non-rural)

“(I’d like to) have an appointment more often than every 6 months, maybe every 3-4 months instead… I’d have more of a sense of accountability…. If I had to meet sooner than every 6 months.” – Participant 6 (65 year old white female, non-rural)

Some of the participants specified that they wanted additional interactions with physicians. Participants identified that many of the follow-up visits are staffed by non-physician practitioners.

“The only difficult part was that I only saw the physician one time, and that was at intake. Everything from there on out was with a [Advanced Practice Practitioner]. So there wasn’t really any assessment data. Only in terms of the scale.” … “In the long term, more frequent benchmarks with the physicians would have been helpful.” – Participant 1 (38 year old white female; non-rural)

Many patients expressed a desire to have more frequent access to specialty staff available at the clinic such as the dietitian, health coach, and bariatric psychologists. Many of them expressed that greater access to specialty providers in the future would potentially enhance their progress.

“When I first started, I was offered psychology support for binge eating, but that was only 3 sessions…. Maybe more psychological help other than just 3 binge eating appointments in the very beginning. Because you know those 3 visits? Well, it’s been like a year and a half now. And those are like long forgotten.” – Participant 2 (63 year old white female; non-rural).

“Yeah. More access to the dietician. I haven’t accessed her since the 1st appointment. I had been scheduled, and then it was rescheduled. And then I think I had something happen. It basically just was like, “Oh, well, just forget it.” By that time I had kinda gotten into a routine and I would guess that she’s probably busy. So you know I wasn’t upset, but I think that would have been helpful to have more access to a dietician.” – Participant 7 (41 year old white female; rural)

### Participant perception of non-judgmental support

Participants expressed that the clinic provided a judgement-free setting surrounding their treatment of obesity. The participants found the clinic to be a space where one could discuss their own weight and weight history in relation to their weight loss goals without stigma.

“They’re just all understanding … I guess they see a lot of people, and they understand the struggles they go through… and they let me know that I’m going through the same thing… and that it’s all right, and we’ll work on it to get it straightened around.” – Participant 6 (65 year old white female; non-rural)

Several participants expressed that they had been hesitant to engage in weight management programs in the past due to the social stigma associated with obesity. Some had explicitly experienced stigma or discrimination from healthcare providers in the past.

“You know, I was hesitant to join, I guess, because I was worried about how I would be seen.” – Participant 10 (51 year old white female; non-rural)

When asked about her interactions with a provider participants described not feeling judged about their weight.

“Even though she was of a typical weight, she wasn’t like super judgy and like acknowledged that it’s difficult…I know I keep saying super judgy, but I don’t know how often you deal with health care providers, but some of them can be not so nice. …dealing with the dietician where I really thought I was going to take some lumps. She was not like that at all. She was really understanding.” – Participant 14 (53 year old white female; non-rural)

For many of these participants, this setting enabled them to continue working on their goals in contrast to previous attempts where they felt a sense of judgement and this discouraged them from seeking help for weight loss.

“You know, there’s kind of like the stigma of like, you know, you’re cheating, you’re taking this medicine, but that’s not how any of the providers I saw like treated. It was like a medicine for a disorder for a diagnosis and that’s, you know, there was no shame involved at all in anything that I was doing. They were really encouraging, especially with my diet and with the exercise that I was doing and continue to do. So, I mean, they were all just extremely sensitive and thoughtful in the way that they responded, I would say.” – Participant 11 (38 year old white female; non-rural)

“I would say the people make a big difference. Because there was absolutely no judgement. Which sometimes, you know, if you go to see a health coach or a personal trainer, you feel like you’re being judged a little bit because, you know, kind of like how did you let yourself get to this point? And I didn’t have any of that with the people at the clinic at all. It was just very supportive.” – Participant 13 (48 year old white female; rural)

### Perception of clinical support following policy changes affecting treatment coverage

Many of the participants were negatively impacted by changes to their insurance policies that resulted in them losing coverage for weight loss medications that were previously covered. This was especially prevalent for the GLP-1 inhibitors such as semaglutide. Despite this, many of the participants continued to engage in the program.

Reasons for participants remaining in the program in spite of insurance policy changes varied. Some believed that the providers at the clinic would be able to work with the insurance companies to reinstate coverage or would be able to identify alternate medications that would either be affordable or covered.

“My insurance a few months later got wise and said, “Well, you’re not diabetic, so we can’t give you that medicine (Ozempic).” So I’m off of that. But in replacing that he was able to come up with a scheme to take a medication called Wellbutrin along with naltrexone. Those 2 medications are the same as the formulary for Contrave, which is a weight loss medicine. Yeah, my insurance won’t pay for Contrave but the components are the two other medicines which he was able to prescribe for me. So I’m still taking those today.” – Participant 3 (68 year old white male; non-rural)

“In talking to the ladies that I work with at the clinic, it sounds like they’ve been advocating with [public employee insurance company] to try to get them to continue to approve it. Kind of like look at the short-term cost versus the long-term benefit, but I don’t think [public employee insurance company] is trying to hear that. So I don’t think that it’s due to lack of trying on their part… But the fact that when I brought it up (the insurance issue), they already knew about it and were already in communication. Like that was a big deal to me and made me feel really positive about their investment in my progress. that they were willing to go to bat.” – Participant 14 (53 year old white female; non-rural)

Other participants believed that the services provided were valuable enough to continue participation regardless of medication usage.

“I’ve just had a really positive experience and I am going to try to continue with them even though I’m trying to figure out if I’m going to pay for that every month or not. I’m planning on at least for the next probably six months, kind of keep going until I figure out what’s going to be next so I feel comfortable enough in doing that.” – Participant 14 (53 year old white female; non-rural)

For those who ultimately did leave the program after insurance policy changes, most only did so as a last resort and did not blame the program itself for any difficulties experienced in the process.

“Overall. I was very happy with the clinic. I just ran out of options after I couldn’t obtain the medication anymore because the savings card had run out and my insurance wouldn’t cover it. I gained back the weight I had lost and that’s when I went back to my primary care provider and said, “Okay, what are next steps, and they referred me to the bariatric clinic.” – Participant 1 (38 Year Old White Female; non-rural)

There were a number of participants who felt that the program did not have much to offer them once medications were no longer an option due to insurance refusing to cover them.

“It was difficult, after I was unable to obtain the medication, because we didn’t really discuss how to maintain the weight loss, or what the next steps would be, or what other options for medications would be. It was kind of like, okay, well, the GLP-1 injectable medications are no longer covered by your insurance. As for next steps, there really weren’t any. So that was definitely a challenge of what to do next in terms of how to maintain the weight loss.” – Participant 1 (38 Year Old White Female; non-rural)

### Participation due to intrinsic motivation

We asked each participant what their motivations were for joining the program. None of them explicitly stated that they were primarily motivated to improve their physical appearance and most were instead motivated by a desire to improve their health.

“I was diagnosed with breast cancer. And my journey through that was complicated. And I had good outcomes. But I had gained weight. And I need to lose that weight to prevent cancer. Before that I knew I needed to lose weight, and you know I had a really bad image. But this was just like a slap in the face. For my help, for health, to prevent cancer recurrence.” – Participant 2 (63 year old white female; non-rural)

“My sister-in-law died because of just horrible obesity and related diseases. And I was like, wow, I do not want to end up like that. And although I was not even close, it was a kind of a wake up call that my health starts now, not when I’m like, you know, 65” – Participant 10 (51 year old white female; non-rural)

There was an even mix of participants who were primarily intrinsically motivation and participants who were motivated by other individuals such as family, friends, or co-workers.

“My 2 sons. I was a single mother. I raised them now they’re 20. Well, 24, and 26. So you know, I was diagnosed with breast cancer and yeah, so definitely, I want to be healthy for them. Because during my cancer journey, of course, I was a single mother, and during my cancer journey I unexpectedly lost my mother the 1st year, and then right when I finished it was complicated. I had 7 surgeries. All the chemo, all the radiation. And then I unexpectedly lost my father. So my sons lost both of their grandparents. And I’m truly all they have left. So yeah.” – Participant 2 (63 year old white female; non-rural)

“It’s mostly me. I have my significant other; he is motivating in some ways but in some ways, he doesn’t think I eat enough. But in order to keep the weight down and to stay on this program, you have to eat small meals throughout the day in order to do that.” – Participant 20 (49 year old white female; non-rural) when asked about who is motivating them.

## Discussion

Obesity is a disease that affects a significant percentage of the population [2,12] and is associated with chronic disease comorbidities [13]. The treatment of obesity is a rapidly evolving field of medicine as highly effective medications and surgical treatment options are developed and approved for use in clinical practice. However, understanding patients’ perspectives when determining the acceptability of a medical clinical practice for obesity is crucial for further defining the needs in this population. In this study, we examined the acceptability of an academic medical weight management clinic through virtual interviews. After analyzing the interview content, five themes emerged, including: 1. Satisfaction with clinical and support staff, and 2. Desire for additional touchpoints with clinical staff, 3. Perception of non-judgmental support, 4. Perception of clinical support following policy changes affecting treatment coverage, and 5. Participation due to self-motivation. These outcomes have several implications for the development of a clinical practice in medical weight management.

The participants reported satisfaction with clinical and support staff. Previous studies have reported similar findings, with most participants in weight loss programs reporting positive experiences overall [9]. Participant satisfaction is important because a positive association between satisfaction with provider lead care and weight loss results even after controlling for baseline characteristics such as starting weight and participant income is correlated [14]. In particular, patients tend to have greater satisfaction with medical or surgical weight loss methods when compared to behavioral weight management methods [15]. Regardless of the weight loss method, the same study found that the amount of weight lost was always the most important criteria for satisfaction [16]. Some studies have found that satisfaction with interventional weight management methods tends to decline with time [17]. Most of the participants in this study had been attending the clinic for an average of over two years and still retained satisfaction, which is incongruent with this previous work.

Participants in this study expressed a desire for more touchpoints, which may have possible implications for their future weight loss goals. Multiple studies have evaluated the optimal number of touchpoints for achieving weight loss. Many organizations recommend that programs offer high-intensity appointments starting every week for the first month, then biweekly for months two through six and monthly thereafter for a year [18]. The United States Preventive Services Taskforce recommends 12 sessions in the first year of treatment [19]. Additionally, multiple studies from community settings indicate that less frequent monitoring appointments may also be effective [20, 21]. However, multiple studies have noted that increasing the number of visits does result in a higher likelihood of clinically significant weight loss [20, 21] or increased weight loss [22].

Another significant finding of this study is that participants did not feel stigmatized by the healthcare team. Healthcare stigma is an entity well-known to have a widespread impact on the physical and psychological health of patients with obesity. Weight stigma can be defined as the devaluation of an individual or group due to weight or body size, and affects more than 50% of all persons [23]. Multiple studies have shown increased rates of hypertension, hyperglycemia, diabetes, poorer glycemic control, metabolic syndrome, depression, anxiety, and binge eating disorder in patients exposed to healthcare stigma [23–25]. In addition to increasing the risk of these conditions, recent studies suggest that exposure to weight stigma may exacerbate the disease of obesity itself by triggering physiological and behavioral changes that worsen metabolic health and lead to further weight gain [25]. Stress experienced by individuals subjected to weight stigma leads to activation of the hypothalamic pituitary axis, thus leading to increased cortisol levels, which is a driver for abdominal obesity, hyperglycemia, and metabolic syndrome. Markers of oxidative stress and inflammation are also found to be higher in individuals subjected to weight stigma [ ]. Non-judgmental treatment is critical in the management of patients with obesity to reduce these complications and to prevent additional stress that is weight promoting. Furthermore, patients subjected to stigma are more likely to delay or avoid medical care, have their clinical concerns discounted or dismissed, and have decreased trust in their healthcare providers [23–26].

Many patients feel that the loss of medication coverage through insurance policy-level decisions is stigmatizing [27]. Prior to this study, many participants were affected by changes in prescription coverage. Those interviewed felt both supported and satisfied with the staff despite treatment barriers. One of the biggest challenges that many of the participants interviewed faced was difficulty with getting their insurance to cover their medications [27]. Studies show that most patients respond to insurance changes negatively with hopelessness and fear being common reactions [27]. Additionally, patients with chronic diseases like obesity utilize primary care services less frequently after insurance disruptions and are more likely to be hospitalized for their chronic illnesses [28]. Previous work has found that patients derive emotional support from clinical practices though multiple methods including showing kindness, providing a clinical interaction that centers around listening, and paying attention to social connection in the process of treatment [29]. Despite these challenges with policy effects on clinical care, none of our participants expressed any dissatisfaction with the weight management clinic. Some even opted to continue with the program despite the changes.

Continuity of care is crucial for improving patient health. Previous research has demonstrated that continuity of care is associated with improved patient outcomes [30]. Most patients do consider continuity of care to be important when it comes to their care [31] which would explain why our participants chose to stay with the program after insurance changes. However, despite rating continuity of care as necessary, most patients are hesitant to spend additional time or resources to maintain it [31]. Due to the unpredictable nature of healthcare policy and the costs associated with anti-obesity medications, a large portion of patients in weight management programs will inevitably struggle with obtaining coverage. Programs should develop plans to engage patients despite policy-related changes.

This study noted that participants reported intrinsic motivation to participate in the clinic, driven by a desire to improve their health. There currently exists the perception that patients who take GLP-1 agonists for weight loss desire a particular body type due to vanity. There is mixed evidence to support this conception. One previous survey study examined the motivations for weight loss among young adults and older adults enrolled in a behavioral weight management program, finding that improved physical appearance was a critical motivator for younger adults [32]. This was not the case for older adults in the study. Another study found that the majority of patients interviewed at a free community-based weight loss program were mostly extrinsically motivated, such as by social desirability or financial incentives [33]. While many of our participants mentioned that they were grateful for any perceived improvements in their physical appearance as a result of their weight loss, the majority of participants were intrinsically motivated to improve their health. The lack of importance with regards to physical appearance as a motivating factor aligns with other acceptability studies on medical weight management programs [9] where the majority of individuals interviewed did not mention physical appearance as a motivating factor for their participation. Previous work has found that participants driven by improved physical appearance had reduced success compared to those driven by other motivations, such as health [32]. Additionally, patients with higher levels of intrinsic motivation were more likely to adhere to exercise recommendations in the long term [34]. In general, patients with obesity attempting weight loss are poorly motivated on average due to perceptions of stigma and perceptions of poor support from providers [35]. However, the additional support provided by a medical weight management program and the possible non-response bias from poorly motivated patients [36] may explain why our participants overwhelmingly expressed high levels of motivation. Overall, the participants in our study were both highly motivated and primarily intrinsically motivated. Finding ways to improve participant motivation may be beneficial to patient outcomes and success.

This study has a few limitations. We have included twenty participants in an academic weight management program, which could lead to a biased sample. Former patients who were no longer participating in the program may have also been less likely to respond to interview requests. Also, the majority of our participants expressed satisfaction with the care they received. Nonrespondents to medical satisfaction surveys tend to have lower levels of satisfaction [36] which may have biased our results. Another limitation is that the principal investigator of the study is also a clinician in the population being studied, which may lead to possible social desirability bias. The clinic serves a predominantly rural population however sixty percent of participants did not identify as being from rural locations however this was a self-report variable and we suspect some error in identification of rural or non-rural locations. Location-specific information was not collected, and therefore it is unknown if they were from rural locations. Participants in this study are all seen in a single academic weight management clinic, so the results may not be generalizable to other clinical settings.

### Conclusion

Five core themes were identified in this work: satisfaction with clinical and support staff, desire for additional touchpoints with clinical staff, perception of non-judgmental support, perception of support by the clinic following policy changes affecting treatment coverage, and participation due to intrinsic motivation. From these themes, we can derive multiple ways to potentially improve patient satisfaction and weight loss results for those enrolled in medical weight management clinics. These include minimizing stigma within organizational culture, ensuring an adequate provider-to-patient ratio and accessible specialty staff (dieticians, PT, psychology, etc), having the skill to work around insurance issues, and having providers skilled in both generating as well as harnessing intrinsic motivation. Future directions include interviewing a sample of participants composed entirely of former patients (rather than a mix of current and former patients) to better ascertain negative feedback.
