# Supplementary material for: Barriers, motivations, and understanding: A qualitative study on the acceptability of an academic weight management program
Source: PLoS One. 2026 Jun 10;21(6):e0351217. doi: 10.1371/journal.pone.0351217 (PMC13252745; doi:10.1371/journal.pone.0351217)
Supplement: Attachment — Submitted filename: Response to Reviewers FINAL.docx [file pone.0351217.s002.docx]

Dear PLOS ONE Editors.

Re: PONE-D-25-40184

We sincerely thank the Editor and reviewers for their thoughtful, constructive feedback on our manuscript. We appreciate the time and care taken to provide detailed comments, which have significantly strengthened the clarity, rigor, and overall quality of the work. We have carefully considered each suggestion and have revised the manuscript accordingly. Below, we provide a point-by-point response detailing how each comment was addressed, with corresponding changes clearly indicated in the revised manuscript.

Sincerely,

Treah Haggerty MD

**Reviewer #1:** This is a well written and clear manuscript detailing qualitative results of an acceptability study of a medical weight management program in West Virginia, US. I have no specific comments about the methods, study design or discussion/implications of the study. My only concern has been raised in the limitations of the paper, that there may be a conflict of interest between the lead investigator as a clinician in the program and the generalizability of the results of this acceptability study outside West Virginia, or indeed, outside this individual group of participants in the study.

**Response:** Thank you for the feedback. We agree that there could be a COI with the lead investigator being a clinician in the program and have included this in the limitations of the study (Lines 466-468). Interviews were conducted by research team members that were not a part of the clinic and analysis was also completes with these members also. Emergent themes were discussed amongst the analysis team hoping to also mitigate some of the limitation.

**Reviewer #2:** The topic is timely and important, given the focus on effective obesity management and the unique challenges faced by rural populations. However, I do have some questions/comments as follows:

**Reviewer #2, Comment 1.** In the introduction section, it will be interesting to have the authors clearly state the obesity prevalence rate differences between male and female participants.

**Response:** We have added this to the introduction section (Line 57-59).

**Reviewer #2, Comment 2.** In lines 70 and 76 in the introduction section, there appears to be no connection between the discussion on clinic demand (Line 70) and the shift to adaptability studies (line 76). Can the authors improve the flow of the narrative?

**Response:** We agree that this flow was very choppy. Thank you for this suggestion. We have edited the paragraph to improve the information and flow of the paragraph. (lines 71-79)

**Reviewer #2, Comment 3.** The authors should justify the choice of their participants.

**Response:** We have added this to the procedures under the methods section. (Lines 112-114)

**Reviewer #2, Comment** 4. The authors should also clearly include the exclusion criteria. Also, based on the inclusion criteria presented in the study by the authors, I can see that the criteria were not fully defined based on the Table presented in their work on the participants. E.g.,
a. participants from rural or non-rural communities
b. The authors did not also address demographic diversity, socioeconomic status

**Response:** We added a sentence to address exclusion criteria (Lines 96-98). Rural/non-rural status was not factored into our inclusion/exclusion criteria. When possible, recruitment for this study was done with the intent of gathering a diverse participant population that best represented the diversity within the population served by the clinic.

**Reviewer #2, Comment 5.** Can the authors clarify the date set for the inclusion criteria and the date set for the recruitment period? In its current form, it is confusing.

**Response:** Patients were eligible for recruitment if they had attended an appointment with the clinic at any point in the calendar year of 2023 and were over the age of 18 at the time of recruitment. Participant recruitment took place between 9/1/24 and 11/30/24 (added to lines 106-107). As an example, a 35 year old who attended an appointment on 7/1/23 would have been eligible for recruitment into the study starting 9/1/24.

**Reviewer #2, Comment 6.** In using the transcription from Zoom, can the authors indicate how they addressed the accuracy of the transcription?

**Response:** This transcription service displayed transcribed sentences in real-time within seconds of the conversation occurring. Interviewers were able to make immediate judgments regarding the accuracy of the transcription and would make notes regarding any errors in the transcription for later edits. Additionally, interviewers would review the transcript for accuracy immediately after the conclusion of the interview. (Line 132)

**Reviewer #2, Comment 7.** The authors should indicate how long the interview lasted.

**Response:** Interviews generally took around 20-30 minutes to complete. This information has been added to the methods section under lines 123-132.

**Reviewer #2, Comment 8.** Can the authors further clarify how the content analysis process was carried out? In its current state, it is too brief.

**Response:** This has been expanded. Thanks for this feedback. Lines 136-147.

**Reviewer #2, Comment 9.** In referring to the participants, kindly indicate it as follows: e.g., Participant 1, white-female, Rural. This will help readers to appreciate the quotes.

**Response:** We have modified the attributions of each quotation as per the format requested.

**Reviewer #2, Comment 10.** Kindly attach the key questions used in the interview guide as an appendix. This is to aid in the replicability of the study.

**Response:** We have attached the interview guide given to interviewers.

**Reviewer #2, Comment 11** The authors should remove the colon in front of participants, procedures, data collection, results, data analysis, discussion, and conclusion. Make them unique headings.

**Response:** We have removed the colons behind each heading in the body as per the reviewer’s request.
