# Supplementary material for: Barriers, motivations, and understanding: A qualitative study on the acceptability of an academic weight management program
Source: PLoS One. 2026 Jun 10;21(6):e0351217. doi: 10.1371/journal.pone.0351217 (PMC13252745; doi:10.1371/journal.pone.0351217)
Supplement: Attachment — Submitted filename: Response 3-10-26.docx [file pone.0351217.s003.docx]

Dear PLOS ONE Editors.

Re: PONE-D-25-40184

Thank you for taking the time to help our team improve this manuscript prior to publication. We have addressed each suggestion and concern below.

Sincerely,

Treah Haggerty MD

1. In the abstract section, kindly indicate the number of participants involved in the interview.
   a. **We have added this information to the “methods” section of the abstract. See line 35.**
2. The authors indicate that the participants were recruited via a portal message, but can they clarify how many participants were invited to participate, as this will help provide a better context to appreciate the sample size and potential biases?
   a. **We have added additional information regarding this process. See line 99-100.**
3. Also, the authors indicate that participants were selected on a first-come, first-served basis, but do not mention the efforts they put in place to ensure diversity. The authors need to clarify how diversity was ensured or whether specific demographic quotas were set.
   a. W**e made efforts to interview additional male participants after it was observed that our initial set of interviews were with disproportionately female participants. See lines 109-111 for this information.**
4. The interview questions should please be attached as an appendix.
   a. **We have attached the interview questions as an appendix as per reviewer request.**
5. Were any software or tools used for the coding and analysis?
   a. **No specific software or tools were used in the coding and analysis. We coded the transcripts in Word and utilized comments and highlighting to separate thematic color coding.**
6. Can the authors clarify how the rural and non-rural participants were defined or verified?
   a. **Rural status was determined by self-reporting; we were not permitted by the conditions of our institution’s IRB to collect more precise geographical data.**
7. Were the questions used for the interview guide adopted from a prior study?
   a. **The interview questions were not adopted from a prior study however we did use the Consolidated Framework for Implementation Research, often states as CFIR, to build the interview guide. This is cited on lines 117 to 118. CFIR does include interview guide building tools.**
8. With the interview guide design, were there any piloting? If yes, how was it carried out, and did it inform the final design of the interview guide?
   a. **No piloting was carried out for the final design of the interview guide however interviews were reviewed regularly with the research team to identify any necessary changes as the study progressed. The interview guide was not changed throughout the study.**
