# Supplementary material for: Barriers, motivations, and understanding: A qualitative study on the acceptability of an academic weight management program
Source: PLoS One. 2026 Jun 10;21(6):e0351217. doi: 10.1371/journal.pone.0351217 (PMC13252745; doi:10.1371/journal.pone.0351217)
Supplement: Peer review history

# Author comment: Author response to Decision Letter 0

**DOI:** 10.1371/journal.pone.0351217.r001

**Transfer Alert**

This paper was transferred from another journal. As a result, its full editorial history (including decision letters, peer reviews and author responses) may not be present.

21 Aug 2025

# Peer review: Decision Letter 0

**DOI:** 10.1371/journal.pone.0351217.r002

**Authors:** Godwin Akrong

7 Dec 2025

PONE-D-25-40184

Barriers, Motivations, and Understanding: A qualitative study on the acceptability of an academic weight management program

PLOS ONE

Dear Dr. Haggerty,

Thank you for submitting your manuscript to PLOS ONE. After careful consideration, we feel that it has merit but does not fully meet PLOS ONE’s publication criteria as it currently stands. Therefore, we invite you to submit a revised version of the manuscript that addresses the points raised during the review process.

- **I encourage you to address Reviewer #2’s comments and make the necessary revisions, particularly in improving the introduction and methodology. I look forward to reviewing your revised manuscript.**<o:p></o:p>

-->Please submit your revised manuscript by Jan 21 2026 11:59PM. If you will need more time than this to complete your revisions, please reply to this message or contact the journal office at plosone@plos.org. When you're ready to submit your revision, log on to https://www.editorialmanager.com/pone/ and select the ’submissions Needing Revision' folder to locate your manuscript file.

Please include the following items when submitting your revised manuscript:

- A rebuttal letter that responds to each point raised by the academic editor and reviewer(s). You should upload this letter as a separate file labeled 'Response to Reviewers'.
- A marked-up copy of your manuscript that highlights changes made to the original version. You should upload this as a separate file labeled 'Revised Manuscript with Track Changes'.
- An unmarked version of your revised paper without tracked changes. You should upload this as a separate file labeled ’manuscript'.

If you would like to make changes to your financial disclosure, please include your updated statement in your cover letter. Guidelines for resubmitting your figure files are available below the reviewer comments at the end of this letter.

If applicable, we recommend that you deposit your laboratory protocols in protocols.io to enhance the reproducibility of your results. Protocols.io assigns your protocol its own identifier (DOI) so that it can be cited independently in the future. For instructions see: https://journals.plos.org/plosone/s/submission-guidelines#loc-laboratory-protocols. Additionally, PLOS ONE offers an option for publishing peer-reviewed Lab Protocol articles, which describe protocols hosted on protocols.io. Read more information on sharing protocols at https://plos.org/protocols?utm_medium=editorial-email&utm_source=authorletters&utm_campaign=protocols.

We look forward to receiving your revised manuscript.

Kind regards,

Godwin Banafo Akrong, Ph.D.

Academic Editor

PLOS ONE

**Journal Requirements:**

1. When submitting your revision, we need you to address these additional requirements.

Please ensure that your manuscript meets PLOS ONE’s style requirements, including those for file naming. The PLOS ONE style templates can be found at

https://journals.plos.org/plosone/s/file?id=wjVg/PLOSOne_formatting_sample_main_body.pdf and

https://journals.plos.org/plosone/s/file?id=ba62/PLOSOne_formatting_sample_title_authors_affiliations.pdf

2. In the online submission form, you indicated that “Data can be accessed by contacting the corresponding author.”

All PLOS journals now require all data underlying the findings described in their manuscript to be freely available to other researchers, either a. In a public repository, b. Within the manuscript itself, or c. Uploaded as supplementary information.

This policy applies to all data except where public deposition would breach compliance with the protocol approved by your research ethics board. If your data cannot be made publicly available for ethical or legal reasons (e.g., public availability would compromise patient privacy), please explain your reasons on resubmission and your exemption request will be escalated for approval.

3. If the reviewer comments include a recommendation to cite specific previously published works, please review and evaluate these publications to determine whether they are relevant and should be cited. There is no requirement to cite these works unless the editor has indicated otherwise.

[Note: HTML markup is below. Please do not edit.]

Reviewers' comments:

Reviewer’s Responses to Questions

**Comments to the Author**

1. Is the manuscript technically sound, and do the data support the conclusions?

The manuscript must describe a technically sound piece of scientific research with data that supports the conclusions. Experiments must have been conducted rigorously, with appropriate controls, replication, and sample sizes. The conclusions must be drawn appropriately based on the data presented.

Reviewer #1: Yes

Reviewer #2: Partly

**********

2. Has the statistical analysis been performed appropriately and rigorously?

Reviewer #1: N/A

Reviewer #2: N/A

**********

3. Have the authors made all data underlying the findings in their manuscript fully available?

The PLOS Data policy requires authors to make all data underlying the findings described in their manuscript fully available without restriction, with rare exception (please refer to the Data Availability Statement in the manuscript PDF file). The data should be provided as part of the manuscript or its supporting information, or deposited to a public repository. For example, in addition to summary statistics, the data points behind means, medians and variance measures should be available. If there are restrictions on publicly sharing data—e.g. participant privacy or use of data from a third party—those must be specified.

Reviewer #1: Yes

Reviewer #2: No

**********

4. Is the manuscript presented in an intelligible fashion and written in standard English?

PLOS ONE does not copyedit accepted manuscripts, so the language in submitted articles must be clear, correct, and unambiguous. Any typographical or grammatical errors should be corrected at revision, so please note any specific errors here.

Reviewer #1: Yes

Reviewer #2: Yes

**********

5. Review Comments to the Author

Please use the space provided to explain your answers to the questions above. You may also include additional comments for the author, including concerns about dual publication, research ethics, or publication ethics. (Please upload your review as an attachment if it exceeds 20,000 characters)

**Reviewer #1:** This is a well written and clear manuscript detailing qualitative results of an acceptability study of a medical weight management program in West Virginia, US. I have no specific comments about the methods, study design or discussion/implications of the study. My only concern has been raised in the limitations of the paper, that there may be a conflict of interest between the lead investigator as a clinician in the program and the generalisability of the results of this acceptability study outside West Virginia, or indeed, outside this individual group of participants in the study.

**Reviewer #2:** The topic is timely and important, given the focus on effective obesity management and the unique challenges faced by rural populations. However, I do have some questions/comments as follows:

1. In the introduction section, it will be interesting to have the authors clearly state the obesity prevalence rate differences between male and female participants.

2. In lines 70 and 76 in the introduction section, there appears to be no connection between the discussion on clinic demand (Line 70) and the shift to adaptability studies (line 76). Can the authors improve the flow of the narrative?

3. The authors should justify the choice of their participants.

4. The authors should also clearly include the exclusion criteria. Also, based on the inclusion criteria presented in the study by the authors, I can see that the criteria were not fully defined based on the Table presented in their work on the participants. E.g.,

a. participants from rural or non-rural communities

b. The authors did not also address demographic diversity, socioeconomic status

5. Can the authors clarify the date set for the inclusion criteria and the date set for the recruitment period? In its current form, it is confusing.

6. In using the transcription from Zoom, can the authors indicate how they addressed the accuracy of the transcription?

7. The authors should indicate how long the interview lasted.

8. Can the authors further clarify how the content analysis process was carried out? In its current state, it is too brief.

9. In referring to the participants, kindly indicate it as follows: e.g., Participant 1, white-female, Rural. This will help readers to appreciate the quotes.

10. Kindly attach the key questions used in the interview guide as an appendix. This is to aid in the replicability of the study.

11. The authors should remove the colon in front of participants, procedures, data collection, results, data analysis, discussion, and conclusion. Make them unique headings.

**********

6. PLOS authors have the option to publish the peer review history of their article (what does this mean?). If published, this will include your full peer review and any attached files.

If you choose “no”, your identity will remain anonymous but your review may still be made public.

**Do you want your identity to be public for this peer review?** For information about this choice, including consent withdrawal, please see our Privacy Policy.

Reviewer #1: No

Reviewer #2: No

**********

[NOTE: If reviewer comments were submitted as an attachment file, they will be attached to this email and accessible via the submission site. Please log into your account, locate the manuscript record, and check for the action link "View Attachments". If this link does not appear, there are no attachment files.]

To ensure your figures meet our technical requirements, please review our figure guidelines: https://journals.plos.org/plosone/s/figures

You may also use PLOS’s free figure tool, NAAS, to help you prepare publication quality figures: https://journals.plos.org/plosone/s/figures#loc-tools-for-figure-preparation.

NAAS will assess whether your figures meet our technical requirements by comparing each figure against our figure specifications.

# Author comment: Author response to Decision Letter 1

**DOI:** 10.1371/journal.pone.0351217.r003

30 Jan 2026

Dear PLOS ONE Editors.

Re: PONE-D-25-40184

We sincerely thank the Editor and reviewers for their thoughtful, constructive feedback on our manuscript. We appreciate the time and care taken to provide detailed comments, which have significantly strengthened the clarity, rigor, and overall quality of the work. We have carefully considered each suggestion and have revised the manuscript accordingly. Below, we provide a point-by-point response detailing how each comment was addressed, with corresponding changes clearly indicated in the revised manuscript.

Sincerely,

Treah Haggerty MD

Reviewer #1: This is a well written and clear manuscript detailing qualitative results of an acceptability study of a medical weight management program in West Virginia, US. I have no specific comments about the methods, study design or discussion/implications of the study. My only concern has been raised in the limitations of the paper, that there may be a conflict of interest between the lead investigator as a clinician in the program and the generalizability of the results of this acceptability study outside West Virginia, or indeed, outside this individual group of participants in the study.

Response: Thank you for the feedback. We agree that there could be a COI with the lead investigator being a clinician in the program and have included this in the limitations of the study (Lines 466-468). Interviews were conducted by research team members that were not a part of the clinic and analysis was also completes with these members also. Emergent themes were discussed amongst the analysis team hoping to also mitigate some of the limitation.

Reviewer #2: The topic is timely and important, given the focus on effective obesity management and the unique challenges faced by rural populations. However, I do have some questions/comments as follows:

Reviewer #2, Comment 1. In the introduction section, it will be interesting to have the authors clearly state the obesity prevalence rate differences between male and female participants.

Response: We have added this to the introduction section (Line 57-59).

Reviewer #2, Comment 2. In lines 70 and 76 in the introduction section, there appears to be no connection between the discussion on clinic demand (Line 70) and the shift to adaptability studies (line 76). Can the authors improve the flow of the narrative?

Response: We agree that this flow was very choppy. Thank you for this suggestion. We have edited the paragraph to improve the information and flow of the paragraph. (lines 71-79)

Reviewer #2, Comment 3. The authors should justify the choice of their participants.

Response: We have added this to the procedures under the methods section. (Lines 112-114)

Reviewer #2, Comment 4. The authors should also clearly include the exclusion criteria. Also, based on the inclusion criteria presented in the study by the authors, I can see that the criteria were not fully defined based on the Table presented in their work on the participants. E.g.,

a. participants from rural or non-rural communities

b. The authors did not also address demographic diversity, socioeconomic status

Response: We added a sentence to address exclusion criteria (Lines 96-98). Rural/non-rural status was not factored into our inclusion/exclusion criteria. When possible, recruitment for this study was done with the intent of gathering a diverse participant population that best represented the diversity within the population served by the clinic.

Reviewer #2, Comment 5. Can the authors clarify the date set for the inclusion criteria and the date set for the recruitment period? In its current form, it is confusing.

Response: Patients were eligible for recruitment if they had attended an appointment with the clinic at any point in the calendar year of 2023 and were over the age of 18 at the time of recruitment. Participant recruitment took place between 9/1/24 and 11/30/24 (added to lines 106-107). As an example, a 35 year old who attended an appointment on 7/1/23 would have been eligible for recruitment into the study starting 9/1/24.

Reviewer #2, Comment 6. In using the transcription from Zoom, can the authors indicate how they addressed the accuracy of the transcription?

Response: This transcription service displayed transcribed sentences in real-time within seconds of the conversation occurring. Interviewers were able to make immediate judgments regarding the accuracy of the transcription and would make notes regarding any errors in the transcription for later edits. Additionally, interviewers would review the transcript for accuracy immediately after the conclusion of the interview. (Line 132)

Reviewer #2, Comment 7. The authors should indicate how long the interview lasted.

Response: Interviews generally took around 20-30 minutes to complete. This information has been added to the methods section under lines 123-132.

Reviewer #2, Comment 8. Can the authors further clarify how the content analysis process was carried out? In its current state, it is too brief.

Response: This has been expanded. Thanks for this feedback. Lines 136-147.

Reviewer #2, Comment 9. In referring to the participants, kindly indicate it as follows: e.g., Participant 1, white-female, Rural. This will help readers to appreciate the quotes.

Response: We have modified the attributions of each quotation as per the format requested.

Reviewer #2, Comment 10. Kindly attach the key questions used in the interview guide as an appendix. This is to aid in the replicability of the study.

Response: We have attached the interview guide given to interviewers.

Reviewer #2, Comment 11 The authors should remove the colon in front of participants, procedures, data collection, results, data analysis, discussion, and conclusion. Make them unique headings.

Response: We have removed the colons behind each heading in the body as per the reviewer’s request.

Attachment Submitted filename: Response to Reviewers FINAL.docx

# Peer review: Decision Letter 1

**DOI:** 10.1371/journal.pone.0351217.r004

**Authors:** Godwin Akrong

4 Mar 2026

PONE-D-25-40184R1

Barriers, Motivations, and Understanding: A qualitative study on the acceptability of an academic weight management program

PLOS One

Dear Dr. Haggerty,

Thank you for submitting your manuscript to PLOS ONE. After careful consideration, we feel that it has merit but does not fully meet PLOS ONE’s publication criteria as it currently stands. Therefore, we invite you to submit a revised version of the manuscript that addresses the points raised during the review process.

- **I encourage you to address the comments raised by Reviewer #2 and make the necessary revisions. I look forward to reviewing your revised manuscript.**

Please submit your revised manuscript by Apr 18 2026 11:59PM. If you will need more time than this to complete your revisions, please reply to this message or contact the journal office at plosone@plos.org. When you're ready to submit your revision, log on to https://www.editorialmanager.com/pone/ and select the ’submissions Needing Revision' folder to locate your manuscript file.

Please include the following items when submitting your revised manuscript:

- A letter that responds to each point raised by the academic editor and reviewer(s). You should upload this letter as a separate file labeled 'Response to Reviewers'.
- A marked-up copy of your manuscript that highlights changes made to the original version. You should upload this as a separate file labeled 'Revised Manuscript with Track Changes'.
- An unmarked version of your revised paper without tracked changes. You should upload this as a separate file labeled ’manuscript'.

-->If you would like to make changes to your financial disclosure, please include your updated statement in your cover letter. Guidelines for resubmitting your figure files are available below the reviewer comments at the end of this letter.

If applicable, we recommend that you deposit your laboratory protocols in protocols.io to enhance the reproducibility of your results. Protocols.io assigns your protocol its own identifier (DOI) so that it can be cited independently in the future. For instructions see: https://journals.plos.org/plosone/s/submission-guidelines#loc-laboratory-protocols. Additionally, PLOS ONE offers an option for publishing peer-reviewed Lab Protocol articles, which describe protocols hosted on protocols.io. Read more information on sharing protocols at https://plos.org/protocols?utm_medium=editorial-email&utm_source=authorletters&utm_campaign=protocols.

We look forward to receiving your revised manuscript.

Kind regards,

Godwin Banafo Akrong, Ph.D.

Academic Editor

PLOS One

**Journal Requirements:**

1. If the reviewer comments include a recommendation to cite specific previously published works, please review and evaluate these publications to determine whether they are relevant and should be cited. There is no requirement to cite these works unless the editor has indicated otherwise.

2. Please review your reference list to ensure that it is complete and correct. If you have cited papers that have been retracted, please include the rationale for doing so in the manuscript text, or remove these references and replace them with relevant current references. Any changes to the reference list should be mentioned in the rebuttal letter that accompanies your revised manuscript. If you need to cite a retracted article, indicate the article’s retracted status in the References list and also include a citation and full reference for the retraction notice.

[Note: HTML markup is below. Please do not edit.]

Reviewers' comments:

Reviewer’s Responses to Questions

**Comments to the Author**

1. If the authors have adequately addressed your comments raised in a previous round of review and you feel that this manuscript is now acceptable for publication, you may indicate that here to bypass the “Comments to the Author” section, enter your conflict of interest statement in the “Confidential to Editor” section, and submit your "Accept" recommendation.

Reviewer #1: All comments have been addressed

Reviewer #2: (No Response)

**********

2. Is the manuscript technically sound, and do the data support the conclusions?

The manuscript must describe a technically sound piece of scientific research with data that supports the conclusions. Experiments must have been conducted rigorously, with appropriate controls, replication, and sample sizes. The conclusions must be drawn appropriately based on the data presented.

Reviewer #1: Yes

Reviewer #2: Yes

**********

3. Has the statistical analysis been performed appropriately and rigorously?

Reviewer #1: Yes

Reviewer #2: N/A

**********

4. Have the authors made all data underlying the findings in their manuscript fully available?

The PLOS Data policy requires authors to make all data underlying the findings described in their manuscript fully available without restriction, with rare exception (please refer to the Data Availability Statement in the manuscript PDF file). The data should be provided as part of the manuscript or its supporting information, or deposited to a public repository. For example, in addition to summary statistics, the data points behind means, medians and variance measures should be available. If there are restrictions on publicly sharing data—e.g. participant privacy or use of data from a third party—those must be specified.

Reviewer #1: No

Reviewer #2: Yes

**********

5. Is the manuscript presented in an intelligible fashion and written in standard English?

PLOS ONE does not copyedit accepted manuscripts, so the language in submitted articles must be clear, correct, and unambiguous. Any typographical or grammatical errors should be corrected at revision, so please note any specific errors here.

Reviewer #1: Yes

Reviewer #2: Yes

**********

6. Review Comments to the Author

Please use the space provided to explain your answers to the questions above. You may also include additional comments for the author, including concerns about dual publication, research ethics, or publication ethics. (Please upload your review as an attachment if it exceeds 20,000 characters)

**Reviewer #1:** (No Response)

**Reviewer #2:** The author(s) have carried out an interesting study; however, I have some concerns:

1. In the abstract section, kindly indicate the number of participants involved in the interview.

2. The authors indicate that the participants were recruited via a portal message, but can they clarify how many participants were invited to participate, as this will help provide a better context to appreciate the sample size and potential biases?

3. Also, the authors indicate that participants were selected on a first-come, first-served basis, but do not mention the efforts they put in place to ensure diversity. The authors need to clarify how diversity was ensured or whether specific demographic quotas were set.

4. The interview questions should please be attached as an appendix.

5. Were any software or tools used for the coding and analysis?

6. Can the authors clarify how the rural and non-rural participants were defined or verified?

7. Were the questions used for the interview guide adopted from a prior study?

8. With the interview guide design, were there any piloting? If yes, how was it carried out, and did it inform the final design of the interview guide?

**********

7. PLOS authors have the option to publish the peer review history of their article (what does this mean?). If published, this will include your full peer review and any attached files.

If you choose “no”, your identity will remain anonymous but your review may still be made public.

**Do you want your identity to be public for this peer review?** For information about this choice, including consent withdrawal, please see our Privacy Policy.

Reviewer #1: No

Reviewer #2: No

**********

[NOTE: If reviewer comments were submitted as an attachment file, they will be attached to this email and accessible via the submission site. Please log into your account, locate the manuscript record, and check for the action link "View Attachments". If this link does not appear, there are no attachment files.]

To ensure your figures meet our technical requirements, please review our figure guidelines: https://journals.plos.org/plosone/s/figures

You may also use PLOS’s free figure tool, NAAS, to help you prepare publication quality figures: https://journals.plos.org/plosone/s/figures#loc-tools-for-figure-preparation.

NAAS will assess whether your figures meet our technical requirements by comparing each figure against our figure specifications.

# Author comment: Author response to Decision Letter 2

**DOI:** 10.1371/journal.pone.0351217.r005

11 Mar 2026

Dear PLOS ONE Editors.

Re: PONE-D-25-40184

Thank you for taking the time to help our team improve this manuscript prior to publication. We have addressed each suggestion and concern below.

Sincerely,

Treah Haggerty MD

1. In the abstract section, kindly indicate the number of participants involved in the interview.

We have added this information to the “methods” section of the abstract. See line 35.

2. The authors indicate that the participants were recruited via a portal message, but can they clarify how many participants were invited to participate, as this will help provide a better context to appreciate the sample size and potential biases?

We have added additional information regarding this process. See line 99-100.

3. Also, the authors indicate that participants were selected on a first-come, first-served basis, but do not mention the efforts they put in place to ensure diversity. The authors need to clarify how diversity was ensured or whether specific demographic quotas were set.

We made efforts to interview additional male participants after it was observed that our initial set of interviews were with disproportionately female participants. See lines 109-111 for this information.

4. The interview questions should please be attached as an appendix.

We have attached the interview questions as an appendix as per reviewer request.

5. Were any software or tools used for the coding and analysis?

No specific software or tools were used in the coding and analysis. We coded the transcripts in Word and utilized comments and highlighting to separate thematic color coding.

6. Can the authors clarify how the rural and non-rural participants were defined or verified?

Rural status was determined by self-reporting; we were not permitted by the conditions of our institution’s IRB to collect more precise geographical data.

7. Were the questions used for the interview guide adopted from a prior study?

The interview questions were not adopted from a prior study however we did use the Consolidated Framework for Implementation Research, often states as CFIR, to build the interview guide. This is cited on lines 117 to 118. CFIR does include interview guide building tools.

8. With the interview guide design, were there any piloting? If yes, how was it carried out, and did it inform the final design of the interview guide?

No piloting was carried out for the final design of the interview guide however interviews were reviewed regularly with the research team to identify any necessary changes as the study progressed. The interview guide was not changed throughout the study.

Attachment Submitted filename: Response 3-10-26.docx

# Peer review: Decision Letter 2

**DOI:** 10.1371/journal.pone.0351217.r006

**Authors:** Godwin Akrong

25 May 2026

Barriers, Motivations, and Understanding: A qualitative study on the acceptability of an academic weight management program

PONE-D-25-40184R2

Dear Dr. Haggerty,

We’re pleased to inform you that your manuscript has been judged scientifically suitable for publication and will be formally accepted for publication once it meets all outstanding technical requirements.

Within one week, you’ll receive an e-mail detailing the required amendments. When these have been addressed, you’ll receive a formal acceptance letter and your manuscript will be scheduled for publication.

An invoice will be generated when your article is formally accepted. Please note, if your institution has a publishing partnership with PLOS and your article meets the relevant criteria, all or part of your publication costs will be covered. Please make sure your user information is up-to-date by logging into Editorial Manager at Editorial Manager® and clicking the ‘Update My Information' link at the top of the page. For questions related to billing, please contact billing support.

If your institution or institutions have a press office, please notify them about your upcoming paper to help maximize its impact. If they’ll be preparing press materials, please inform our press team as soon as possible -- no later than 48 hours after receiving the formal acceptance. Your manuscript will remain under strict press embargo until 2 pm Eastern Time on the date of publication. For more information, please contact onepress@plos.org.

Kind regards,

Godwin Banafo Akrong, Ph.D.

Academic Editor

PLOS One

Additional Editor Comments (optional):

Reviewers' comments:

Reviewer’s Responses to Questions

**Comments to the Author**

1. If the authors have adequately addressed your comments raised in a previous round of review and you feel that this manuscript is now acceptable for publication, you may indicate that here to bypass the “Comments to the Author” section, enter your conflict of interest statement in the “Confidential to Editor” section, and submit your "Accept" recommendation.

Reviewer #2: All comments have been addressed

Reviewer #3: All comments have been addressed

**********

2. Is the manuscript technically sound, and do the data support the conclusions?

The manuscript must describe a technically sound piece of scientific research with data that supports the conclusions. Experiments must have been conducted rigorously, with appropriate controls, replication, and sample sizes. The conclusions must be drawn appropriately based on the data presented.

Reviewer #2: Yes

Reviewer #3: Yes

**********

3. Has the statistical analysis been performed appropriately and rigorously?

Reviewer #2: N/A

Reviewer #3: N/A

**********

4. Have the authors made all data underlying the findings in their manuscript fully available?

The PLOS Data policy requires authors to make all data underlying the findings described in their manuscript fully available without restriction, with rare exception (please refer to the Data Availability Statement in the manuscript PDF file). The data should be provided as part of the manuscript or its supporting information, or deposited to a public repository. For example, in addition to summary statistics, the data points behind means, medians and variance measures should be available. If there are restrictions on publicly sharing data—e.g. participant privacy or use of data from a third party—those must be specified.

Reviewer #2: Yes

Reviewer #3: Yes

**********

5. Is the manuscript presented in an intelligible fashion and written in standard English?

PLOS ONE does not copyedit accepted manuscripts, so the language in submitted articles must be clear, correct, and unambiguous. Any typographical or grammatical errors should be corrected at revision, so please note any specific errors here.

Reviewer #2: Yes

Reviewer #3: Yes

**********

6. Review Comments to the Author

Please use the space provided to explain your answers to the questions above. You may also include additional comments for the author, including concerns about dual publication, research ethics, or publication ethics. (Please upload your review as an attachment if it exceeds 20,000 characters)

Reviewer #2: Thank you for the thorough revisions. The authors have addressed all of my previous comments, and I have no further feedback at this time.

Reviewer #3: The authors have adequately addressed the reviewer comments and the manuscript has improved substantially. I have no major remaining concerns and recommend acceptance.

As a minor editorial suggestion, the authors could briefly clarify in the Methods section that coding was conducted manually in Word without dedicated qualitative analysis software, as this is currently described only in the response letter.

**********

7. PLOS authors have the option to publish the peer review history of their article (what does this mean?). If published, this will include your full peer review and any attached files.

If you choose “no”, your identity will remain anonymous but your review may still be made public.

**Do you want your identity to be public for this peer review?** For information about this choice, including consent withdrawal, please see our Privacy Policy.

Reviewer #2: No

Reviewer #3: No

**********

# Editor report: Acceptance letter

**DOI:** 10.1371/journal.pone.0351217.r007

**Authors:** Godwin Akrong

PONE-D-25-40184R2

PLOS One

Dear Dr. Haggerty,

I’m pleased to inform you that your manuscript has been deemed suitable for publication in PLOS One. Congratulations! Your manuscript is now being handed over to our production team.

At this stage, our production department will prepare your paper for publication. This includes ensuring the following:

* All references, tables, and figures are properly cited

* All relevant supporting information is included in the manuscript submission,

* There are no issues that prevent the paper from being properly typeset

You will receive further instructions from the production team, including instructions on how to review your proof when it is ready. Please keep in mind that we are working through a large volume of accepted articles, so please give us a few days to review your paper and let you know the next and final steps.

Lastly, if your institution or institutions have a press office, please let them know about your upcoming paper now to help maximize its impact. If they'll be preparing press materials, please inform our press team within the next 48 hours. Your manuscript will remain under strict press embargo until 2 pm Eastern Time on the date of publication. For more information, please contact onepress@plos.org.

You will receive an invoice from PLOS for your publication fee after your manuscript has reached the completed accept phase. If you receive an email requesting payment before acceptance or for any other service, this may be a phishing scheme. Learn how to identify phishing emails and protect your accounts at https://explore.plos.org/phishing.

If we can help with anything else, please email us at customercare@plos.org.

Thank you for submitting your work to PLOS ONE and supporting open access.

Kind regards,

PLOS ONE Editorial Office Staff

on behalf of

Dr. Godwin Banafo Akrong

Academic Editor

PLOS One
